# Evaluation of radar-based precipitation estimates during a flood event using rain gauge validation

**DOI:** 10.1038/s41598-026-40456-z

**Published:** 2026-04-02

**Authors:** Karol Dzwonkowski, Ireneusz Winnicki, Sławomir Pietrek, Krzysztof Kroszczyński

**Affiliations:** Institute of Geospatial Engineering and Geodesy, Faculty of Civil Engineering and Geodesy, Military University of Technology, Warsaw, Poland

**Keywords:** Weather radar, Flood event, Quantitative precipitation estimates (QPE), Precipitation accuracy, Rain gauge validation, Climate sciences, Environmental sciences, Hydrology, Natural hazards

## Abstract

This study uses dual-polarization radar data from two weather radars to assess the accuracy of estimating rainfall rate (*R*) from radar reflectivity (*Z*) using classical empirical Z–R relationships (Marshall–Palmer, Wexler, and Doumoulin–Cogombles). Three additional polarimetric relationships incorporating differential reflectivity (ZDR) were also analyzed. Calculations were carried out for seven altitude levels, representing a novel approach to verifying the vertical structure of the precipitation field. Radar data were validated against measurements from a rain gauge network, enabling an assessment of spatiotemporal consistency and rainfall estimation errors. The analysis covers measurements collected during the flood event that occurred in Poland in September 2024 (Genoa low-pressure system). The results indicate that for the analyzed extreme flood event, the most reliable precipitation totals were obtained using the polarimetric relationship based on ZDR (RMSE=2.20 mm±0.90 mm, MAE=1.84 mm±0.73 mm, and Bias=-0.67 mm±0.81 mm). It was demonstrated that under the studied extreme conditions, the proposed ZDR3 relationship exhibits approximately 69% lower Bias compared with the standard operational Marshall-Palmer method. The findings confirm the potential of polarimetric methods for rainfall estimation and their applicability in operational settings during similar extreme weather events, particularly in early warning and crisis management systems.

## Introduction

Intense and long-lasting rainfall, which may lead to flooding and other hazardous extreme events, poses a substantial threat to human life, livestock, and both building and transportation infrastructure. Effective monitoring and forecasting of precipitation therefore require modern observational systems and advanced data-processing techniques^1–9^. Radar measurements play a particularly important role in this context, as they allow real-time analysis of the structure and dynamics of atmospheric phenomena^10–12^. Radar datasets provide critical information on the spatial distribution and intensity of precipitation. By applying appropriate algorithms to convert these observables into rainfall estimates, reliable data can be generated for early warning systems and crisis management centers^13–19^.

Technological advances in meteorological radar systems have significantly enhanced the capability and accuracy of measurements used to detect and analyze atmospheric structures. Modern radar networks now employ dual-polarization technology for volumetric atmospheric scanning^15,16,20–26^. Since 2024, the Polish POLRAD weather radar network has operated ten such advanced systems, acquiring data using a five-minute scanning strategy. This temporal resolution enables continuous monitoring of the synoptic situation and real-time assessment of evolving weather phenomena^27–29^. However, the standard methods used to convert radar reflectivity (*Z*) into rainfall rate (*R*), based on empirical Z–R relationships, remain subject to considerable uncertainties that may result in errors in rainfall estimation.

Algorithms used to estimate precipitation from weather radar observations rely on converting radar reflectivity (*Z*) into rainfall rate (*R*). In operational radar systems, this conversion is typically performed using empirical relationships^30^. The most widely applied formulation is the Marshall–Palmer relationship. However, applying this relationship throughout the year without considering the radar sampling height, current synoptic conditions, and local environmental or orographic factors can lead to substantial estimation errors^4,31–36^. Radar measurements therefore require appropriate calibration and parameterization of the applied relationships. Consequently, numerous studies have evaluated alternative empirical formulations^21,31,32,34,37–40^. In this study, in addition to the Marshall–Palmer relationship, the Wexler and Doumoulin–Cogombles relationships were implemented, each further adjusted using the polarimetric specific differential phase (KDP).

Modern dual-polarization weather radars provide extensive datasets that include polarimetric parameters, which can be used both to identify the type of precipitation and to estimate its intensity^41,42^. One of the key polarimetric variables is differential reflectivity (ZDR), defined as the ratio of horizontally to vertically polarized reflectivity. Proper interpretation of ZDR enables identification of hydrometeor size and supports more accurate characterization of precipitation intensity^15,21,37^. Therefore, in this study, in addition to the three empirical Z–R relationships, rainfall rate was also estimated using three polarimetric relations based on the ZDR parameter, which is particularly relevant for measurements in mountainous terrain.

Monitoring precipitation in mountainous regions poses significant challenges resulting from complex terrain orography and inherent limitations of radar measurements. Under such conditions, appropriate parameterization of atmospheric scanning products becomes particularly important^15,43^. High accuracy in precipitation analyses over areas with complex topography has been demonstrated in studies employing data from two radars, which enables cross-verification of retrieved measurements and allows the influence of radar range on data quality to be taken into account^15,20,44^.

**Fig. 1:**
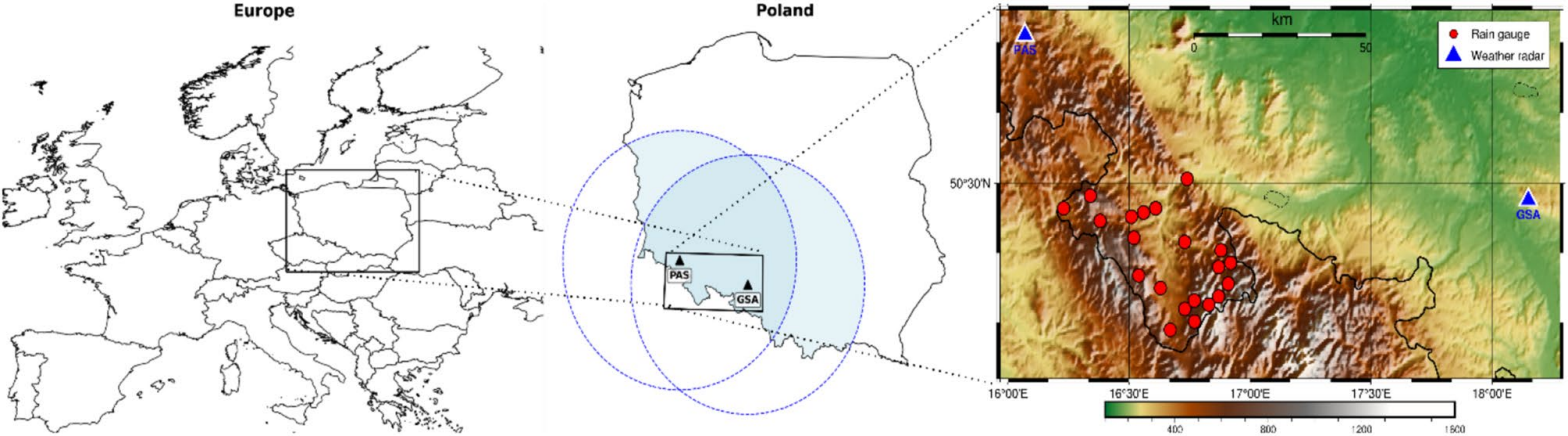
Study area shown at the European, Polish, and local scales, with radar coverage areas and meteorological station locations. The maps were generated by the authors using the Generic Mapping Tools (PyGMT ver. 0.17.0; https://www.generic-mapping-tools.org/). The final multi-panel figure was composed and formatted using draw.io (https://app.diagrams.net/).

Rain gauges are essential for the proper calibration and parameterization of radar measurements, as they provide reference observations for validating radar-derived values and assessing the quality and accuracy of radar-based precipitation estimates^33,41,45–47^. In this study, verification of radar-derived rainfall data was performed using a network of 21 meteorological stations located within the study area (Fig. 1). This number of stations ensures the representativeness of the results by providing adequate spatiotemporal coverage.

Previous studies^48^ focused on analyzing three other empirical relationships: Marshall–Palmer, Joss, and Muchnik using radar data from altitudes of 1 km, 1.5 km, and 2 km. It was shown that, due to the observed underestimation of radar-derived rainfall totals, rainfall rates estimated at 1.5 km using the Marshall–Palmer relationship were the closest to the actual values obtained from rain gauge measurements. In the case of an extensive cloud zone associated with a wind convergence line, during which radar reflectivity reached 55.5 dBZ, the rainfall rate estimated at 1.5 km was as much as 32.51 mm⋅h^-1^ higher than the value calculated using the operationally applied 1 km height. In our subsequent study^49^, verification was carried out using precipitation maps, statistical indicators, and rain gauge observations. The results confirmed that data from 1.5 km, estimated using the Marshall–Palmer relationship, exhibited the lowest mean absolute error (MAE=1.99 mm) and very high agreement with rain gauge measurements (Pearson correlation coefficient r=0.98).

The aim of this study is to evaluate the quality of radar-derived precipitation data relative to ground-based measurements from selected meteorological stations, as well as to analyze the effectiveness of their spatiotemporal matching methods. The paper presents a procedure for integrating radar data with station observations, with particular emphasis on validation aspects. The analysis evaluates rainfall totals using three empirical Z–R relationships: Marshall–Palmer, Wexler, and Doumoulin–Cogombles. These were applied at seven height levels: 1.0 km, 1.2 km, 1.4 km, 1.5 km, 1.6 km, 1.8 km, and 2.0 km above ground level (AGL). Additionally, three polarimetric relationships based on ZDR were assessed at seven reference levels above mean see level (AMSL). Radar data were obtained from two systems (Pastewnik and Góra Św. Anny). This dual-radar approach enables cross-comparison of their outputs and an assessment of how radar range influences estimation quality. Validation of radar-derived precipitation was performed using a network of 21 rain gauges located within the study area, providing representative spatial coverage. A spatiotemporal analysis was conducted to assess the performance of the empirical and polarimetric relationships in precipitation estimation under conditions of complex terrain, and to determine their suitability for operational rainfall monitoring. The accuracy assessment was carried out using commonly applied statistical indicators: root mean square error (RMSE), mean absolute error (MAE), bias, and the Pearson correlation coefficient (*r*).

The results of these studies aim to determine the extent to which radar data can serve as a reliable source of information for meteorological and hydrological applications, operational aviation support, and early warning systems for extreme weather events. Primarily, these methods will be parameterized and validated using in-situ measurements.

## Study area, data, and methodology

### Study area

The study area encompasses the Central and Eastern Sudetes, including the intermontane basins of the Kłodzko Basin and the Ścinawka Trough, as well as the Orlickie Foothills and the surrounding mountain ranges: the Stołowe, Kamienne, and Bystrzyckie Mountains to the northwest and west, and the Śnieżnik Massif and the Golden and Bardzkie Mountains to the east. These physiographic units form the northeastern part of the Bohemian Massif, a tectonic block composed of metamorphic and igneous rocks, younger Paleozoic structures, and sedimentary formations. The Bohemian Massif is characterized by fault-block mountain morphology, with elevations in the study area exceeding 1000 m a.m.s.l. (Śnieżnik, 1425 m a.m.s.l.)^50–52^. In this region, the complex topography plays a crucial role in modulating both the spatial distribution and the intensity of precipitation, particularly during inflow of moist air masses from the south. This makes the area especially relevant for evaluating the accuracy of radar-based precipitation estimates. The region was selected for analysis due to the occurrence of a severe flood event in September 2024, associated with the passage of a Genoa low-pressure system (following the Vb van Bebber track)^51^.

Within the study area, 21 meteorological stations operated by IMWM-NRI are located and were used for validating the radar-derived precipitation estimates. The stations are situated at elevations ranging from 321 m a.m.s.l. to 1218 m a.m.s.l. To improve readability and clearly illustrate the location of each station and radar, a letter–number identifier was assigned to all sites. A summary of station and radar names, geographic coordinates, elevations, and corresponding identifiers is provided in Table 1.

**Table 1:** Overview of meteorological stations and radars, including their IDs, names, coordinates, and elevations.

ID	Station	Coordinates	Elevation [m a.m.s.l.]
S1	Bardo	50°30′33″ N, 16°44′39″ E	321
S2	Karłów	50°28′27″ N, 16°20′13″ E	753
S3	Kudowa-Zdrój	50°26′17″ N, 16°13′19″ E	364
S4	Słoszów	50°24′36″ N, 16°22′46″ E	556
S5	Szalejów Górny	50°25′50″ N, 16°33′09″ E	335
S6	Kłodzko	50°26′13″ N, 16°36′51″ E	356
S7	Polanica-Zdrój	50°25′31″ N, 16°31′06″ E	389
S8	Starkówek	50°22′24″ N, 16°31′05″ E	484
S9	Ołdrzychowice Kłodzkie	50°21′24″ N, 16°44′05″ E	349
S10	Lądek-Zdrój	50°20′43″ N, 16°53′06″ E	461
S11	Spalona	50°16′36″ N, 16°32′18″ E	815
S12	Długopole-Zdrój	50°14′59″ N, 16°38′00″ E	364
S13	Stary Gierałtów	50°18′36″ N, 16°55′03″ E	535
S14	Stronie Śląskie	50°18′09″ N, 16°51′58″ E	502
S15	Bolesławów	50°15′17″ N, 16°53′27″ E	602
S16	Kamienica	50°13′38″ N, 16°53′03″ E	682
S17	Śnieżnik	50°12′30″ N, 16°49′57″ E	1218
S18	Międzygórze	50°13′07″ N, 16°46′25″ E	795
S19	Nowa wieś	50°11′52″ N, 16°44′42″ E	555
S20	Jodłów	50°10′22″ N, 16°46′16″ E	828
S21	Międzylesie	50°09′12″ N, 16°40′15″ E	453
PAS	Radar Pastewnik	50°52′58″ N, 16°02′23″ E	691
GSA	Radar Góra Św. Anny	50°27′44″ N, 18°09′04″ E	433

All meteorological stations are located within the coverage areas of two POLRAD weather radars: Pastewnik and Góra Św. Anny (Table 1). The PAS radar is situated 258 meters higher than the GSA radar, which may influence the results of atmospheric scanning. The locations of the stations, radars, and the study area are shown in Fig. 1.

**Fig. 2:**
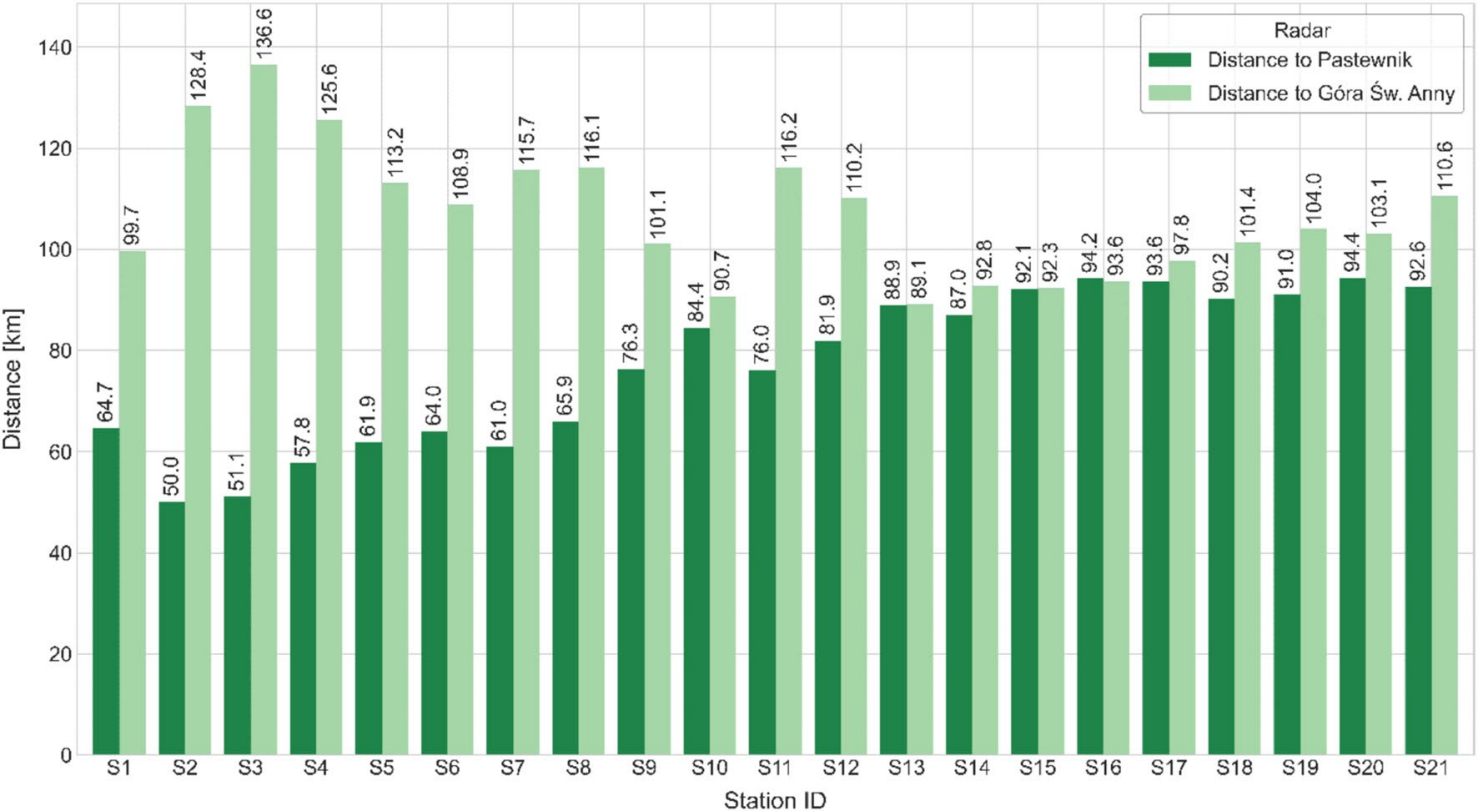
Distances of the meteorological stations from the Pastewnik and Góra Św. Anny radars.

The distance between the meteorological stations and the radars varies considerably (Fig. 2). The shortest distance is 50 km, between the station in Karłów and the Pastewnik radar, while the longest is 136.6 km, between the station in Kudowa-Zdrój and the Góra Św. Anny radar. The average distance of the stations from the Pastewnik radar is 77.1 km, and from the Góra Św. Anny radar approximately 107 km. Most of the stations are located closer to the Pastewnik radar. In this study, the influence of both the radar–station distance and the station elevation above sea level on the accuracy of radar-based precipitation estimates was evaluated.

## Data

This study used data obtained from two types of measurements: remote sensing and in situ observations. Radar data were derived from atmospheric scanning performed by meteorological radars belonging to the POLRAD weather radar network. Hourly precipitation totals were obtained from rain gauge measurements carried out at meteorological stations located within the study area.

Rain gauge measurements represent point observations, whereas radar measurements provide area-averaged information over a larger spatial domain. Although this spatial mismatch presents challenges for direct comparison, it enables a comprehensive assessment of radar data quality. It also allows for the identification of potential local discrepancies that may arise from terrain effects, atmospheric conditions, or the scattering properties of the radar signal.

All rain gauges used in this study are located within the scanning range of both radars, and their distribution was selected to ensure uniform coverage of the analyzed area. The instruments are operated by the Institute of Meteorology and Water Management – National Research Institute (IMWM-NRI), which ensures high data quality and reliability through standardized measurement procedures and regular calibration checks.

Radar data were obtained from two meteorological radars located in Pastewnik and Góra Św. Anny (Fig. 1) and were generated using the RAPOK 5.1.7 and RAINDOW DART 5 software packages (Leonardo), which enable radar data processing, visualization, and quantitative analysis. Both radars operate in the C band and use dual-polarization technology, allowing the use of radar reflectivity (*Z*), differential reflectivity (ZDR), and specific differential phase shift (KDP). Under operational practice, rainfall rate is typically estimated from radar reflectivity at a height of 1 km above ground level. In this study, additional height levels: 1.2 km, 1.4 km, 1.5 km, 1.6 km, 1.8 km, and 2.0 km above ground level and were included to refine the representation of precipitation structure in mountainous terrain. The selection of these heights was based on previous research and the need for detailed mapping of precipitation fields in areas with complex topography.

For values derived using the ZDR parameter, the same height levels were applied; however, they were referenced to mean sea level due to the characteristics of the computational algorithm and the structure of the radar software output. The following radar data parameters were used in the analysis:

- spatial resolution: 1 km × 1 km;
- temporal resolution: 5 minutes;
- C-band electromagnetic wave with horizontal and vertical polarization;
- radar variables:
- radar reflectivity (Z);
- differential reflectivity (ZDR);
- dual-polarization surface rainfall intensity (DPSRI) corrected using specific differential phase (KDP);
- precipitation accumulation (PAC).

Rain gauge data were obtained from the measurement database of IMWM-NRI. The meteorological stations are equipped with rain gauges with a measurement resolution of 0.1 mm. The rain gauge observations served as reference values for validating the radar-derived precipitation estimates. The data underwent a quality control procedure that included:

- selection of meteorological stations representative of the study area;
- temporal and spatial synchronization with the radar data (hourly precipitation totals);
- aggregation of the measurements to hourly precipitation sums.

**Fig. 3:**
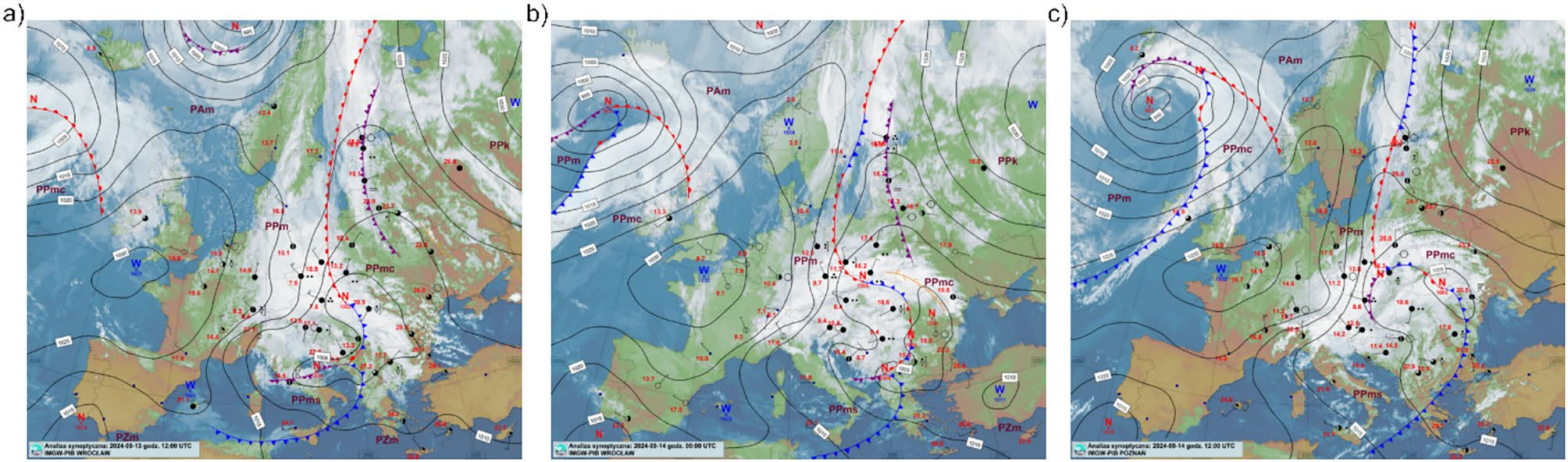
Synoptic situation over Europe. (**a**) 13 September 2024 at 12:00 UTC; (**b**) 14 September 2024 at 00:00 UTC; (**c**) 14 September 2024 at 12:00 UTC. Source: Institute of Meteorology and Water Management – National Research Institute^53^.

During the analyzed period, from 13 September 2024 at 10:00 UTC to 14 September 2024 at 21:00 UTC, the study area was influenced by a low-pressure system (a Genoa low) and its associated atmospheric fronts (Fig. 3). Throughout this period, both stratiform and convective rain occurred, driven by the prevailing synoptic conditions and locally intensified by orographic effects. The total precipitation measured by the rain gauges ranged from 49.4 mm at station S3 to 286.1 mm at station S16.

## Research methodology

**Fig. 4:**
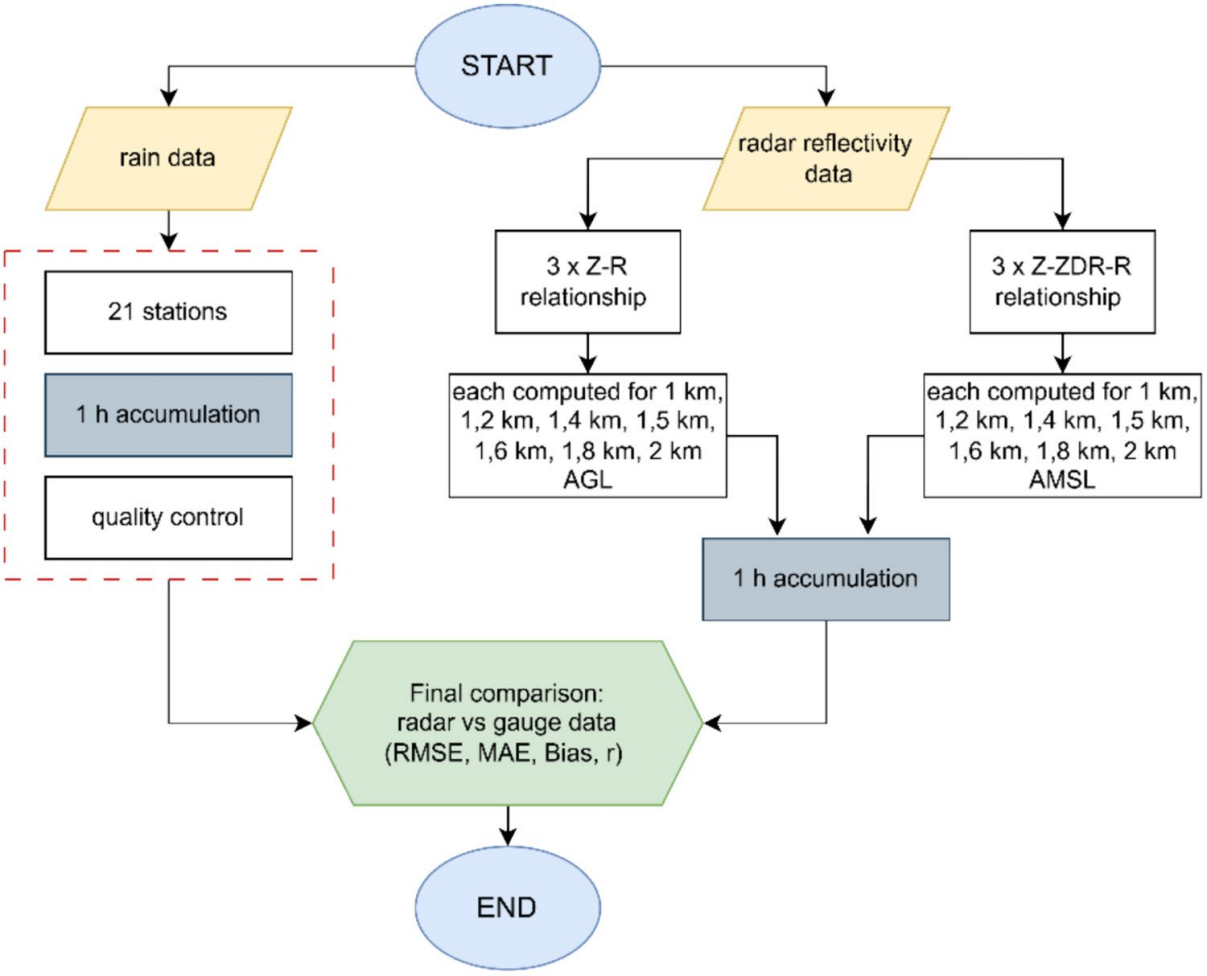
Workflow illustrating the data processing steps and validation procedure used in this study. The process includes the acquisition and preprocessing of radar and rain gauge data, temporal and spatial alignment, estimation of precipitation totals using empirical and polarimetric relationships, and subsequent validation using RMSE, MAE, Bias, and the Pearson correlation coefficient.

The research methodology involved the acquisition, preparation, and processing of radar and rain gauge data. Precipitation totals were estimated from radar-derived rainfall rates using radar reflectivity. Spatiotemporal aggregation of the radar data was performed for the locations of the rain gauge stations, followed by validation of the radar-derived precipitation totals against rain gauge measurements using statistical performance metrics. The data processing workflow is presented in Fig. 4.

To conduct the analysis, it was necessary to obtain hourly precipitation totals from the rain gauge network. Hourly precipitation sums were collected from 21 meteorological stations selected for their representative coverage of the study area. The data underwent quality control and were subsequently aggregated prior to comparison with radar-derived precipitation estimates.

A temporal alignment procedure was applied to harmonize the timing of the radar and rain gauge measurements. The temporal scales of both observation types were standardized, and the validation was performed using hourly precipitation totals derived from the two measurement systems.

Radar-derived precipitation data were prepared using two approaches. The first approach involved the operational method implemented through the RAINBOW DART software. Within this framework, radar data quality control was performed using the RADVOL-QC algorithm, implemented in the Polish radar network (POLRAD). This algorithm executes a multistage preprocessing sequence to eliminate ground clutter and non-meteorological echoes. It also corrects measurement errors before the quantitative precipitation estimation (QPE) stage. To identify terrain-induced and non-meteorological echoes, advanced polarimetric algorithms were employed, based on Doppler filtering and the correlation coefficient (ρ_hv_), which describes the correlation between horizontally and vertically polarized signals. Due to the mountainous terrain, an orography-based algorithm was critical. It corrects for partial and total beam blockage using a Digital Elevation Model (DEM) and beam propagation geometry analysis. Additionally, attenuation in precipitation zones was corrected using polarimetric variables, which is particularly significant for C-band radars. Specifically, the differential propagation phase (Φ _DP_) and the specific differential phase shift (K_DP_) were utilized to improve accuracy^54,55^. A key element in enhancing the reliability of precipitation estimates in this study was the integration of data from two independent radar systems: Pastewnik (PAS) and Góra Św. Anny (GSA). This multi-radar approach facilitated beam blockage compensation and reduced range-dependent errors. It also enabled mutual verification of the radar measurements. According to the algorithms applied in the POLRAD national weather radar system, radar reflectivity (*Z*) measured at a height of 1 km above ground level is converted into rainfall rate (*R*) using the following relationship:

- when *Z*>38 dBZ and KDP>0.15°⋅km^-1^, the following relationship is applied:
  1 $$R=33.5\cdot {|KDP|}^{0.83}$$
- in all other cases, the empirical relationship is used:
  2 $$Z=a\cdot {R}^{b}$$

where:

KDP is the specific differential phase shift of the radar signal,

*a, b* are empirically derived numerical parameters.

For the purposes of this study, rainfall rate was derived from radar reflectivity using the operationally applied procedure. In addition to the standard Marshall–Palmer relationship for data at 1 km above ground level (AGL), the empirical relationships of Wexler and Doumoulin–Cogombles (Table 2) were also applied for heights of 1.2 km, 1.4 km, 1.5 km, 1.6 km, 1.8 km, and 2.0 km above ground level (AGL). Based on the resulting rainfall rate values, hourly precipitation totals were subsequently calculated.

**Table 2:** Overview of the relationships used in the analysis to derive precipitation totals.

Relationship	Equation no.	Equation	ID
Marshall-Palmer	(3)	$Z=200\cdot {R}^{1.6}$	MP
Wexler	(4)	$Z=224\cdot {R}^{1.54}$	W
Doumoulin-Cogombles	(5)	$Z=255\cdot {R}^{1.45}$	D
Used in other study^21^	(6)	$R=0.0144\cdot {Z}^{0.761}\cdot {ZDR}^{-1.51}$	ZDR1
Proposed equations	(7)	$R=0.018\cdot {Z}^{0.82}\cdot {ZDR}^{-1.2}$	ZDR2
	(8)	$R=0.013\cdot {Z}^{0.94}\cdot {ZDR}^{-0.1}$	ZDR3

Given that the radar measurements were performed using dual-polarization technology, a second approach to deriving precipitation totals involved the use of relationships incorporating polarimetric information from differential reflectivity (ZDR). These relationships were applied for heights of 1.0 km, 1.2 km, 1.4 km, 1.5 km, 1.6 km, 1.8 km, and 2.0 km above mean sea level (AMSL). Rainfall rate was estimated using combined radar reflectivity and differential reflectivity relationships (Table 2).

To derive the coefficients for the proposed polarimetric relationships, two distinct approaches were utilized, accounting for both the physical characteristics of precipitation and statistical optimization. The ZDR1 relationship was selected based on established literature^21^, as it had previously been identified as a highly effective algorithm for polarimetric systems. In this study, it serves as a robust benchmark. For the ZDR2 and ZDR3 relationships, a traditional window matching method was applied to pair radar observations with ground-based rain gauge measurements. For each of the 21 meteorological stations, a 5×5 pixel spatial window (corresponding to an area of 25 km^2^) centered on the rain gauge location was defined. This window size was selected due to the complex orography and the need to mitigate errors from horizontal wind drift. Such drift was particularly pronounced during the analyzed Genoa Low event. The use of a 25 km^2^ window allowed for the statistical averaging of local precipitation heterogeneities. This resulted in greater stability compared to smaller areas, which are more susceptible to spatial displacement errors. Prior to calculating the mean values of radar variables Z and ZDR within this window, the data underwent rigorous quality control via the RADVOL-QC system. This facilitated the automatic exclusion of pixels identified as anomalous (e.g., ground clutter or non-meteorological echoes); consequently, the averaging process was based exclusively on reliable meteorological data.

The coefficients for the ZDR2 and ZDR3 equations were derived through local empirical calibration, tailoring them specifically to the analyzed flood event in Poland. This choice was driven by the strong dependence of polarimetric parameters on local cloud microphysics. During an extreme Genoa Low, raindrop size and shape variability may deviate significantly from standard models. This process involved iterative parameter optimization to minimize residuals. Such an approach allowed for the derivation of relationships that directly reflect the characteristics of high-intensity rainfall in complex terrain. The resulting coefficients for all derived relationships are summarized in Table 2.

Aggregation of the radar data into hourly precipitation totals was based on the previously derived rainfall rate values. Using the calculated rainfall rates, hourly accumulations were computed for each pixel of the radar grid. For each meteorological station, the mean value from a 5×5-pixel window surrounding the station location was calculated. Spatial averaging, supported by RADVOL-QC filtering, reduced the influence of isolated anomalies and noise. This procedure ensured that radar-derived totals were representative for comparison with rain gauge observations by aligning the sampling volume with ground-level accumulation.

Statistical metrics were used to compare radar-derived and rain gauge precipitation totals. The evaluation employed the root mean square error (RMSE), mean absolute error (MAE), systematic error (Bias), and the Pearson linear correlation coefficient (*r*). These metrics are based on the rain gauge measurements (*x*), the corresponding radar estimates (*y*), their respective means ($\overline{x } , \overline{y }$​), and the number of observations (*n*)^16,55–57^. Table 3 presents the formulas used to compute the statistical indicators, along with their possible ranges and optimal values.

**Table 3:** Summary of the statistical indicators used for evaluating the results.

Name	Equation	Equation no.	Possible range	Ideal Value
RMSE	$RMSE=\sqrt{\frac{1}{n}\sum {\left(y-x\right)}^{2}}$	(9)	[0, +∞)	0
MAE	$MAE=\frac{1}{n}\sum \left|y-x\right|$	(10)	[0, +∞)	0
Bias	$Bias=\frac{1}{n}\sum (y-x)$	(11)	(-∞, +∞)	0
r	$r= \frac{\sum \left(x-\overline{x }\right)(y-\overline{y })}{\sqrt{\sum {\left(x-\overline{x }\right)}^{2}\sum {(y-\overline{y })}^{2}}}$	(12)	[-1, 1]	1

## Results

### Evaluation of rainfall estimation methods

**Fig. 5:**
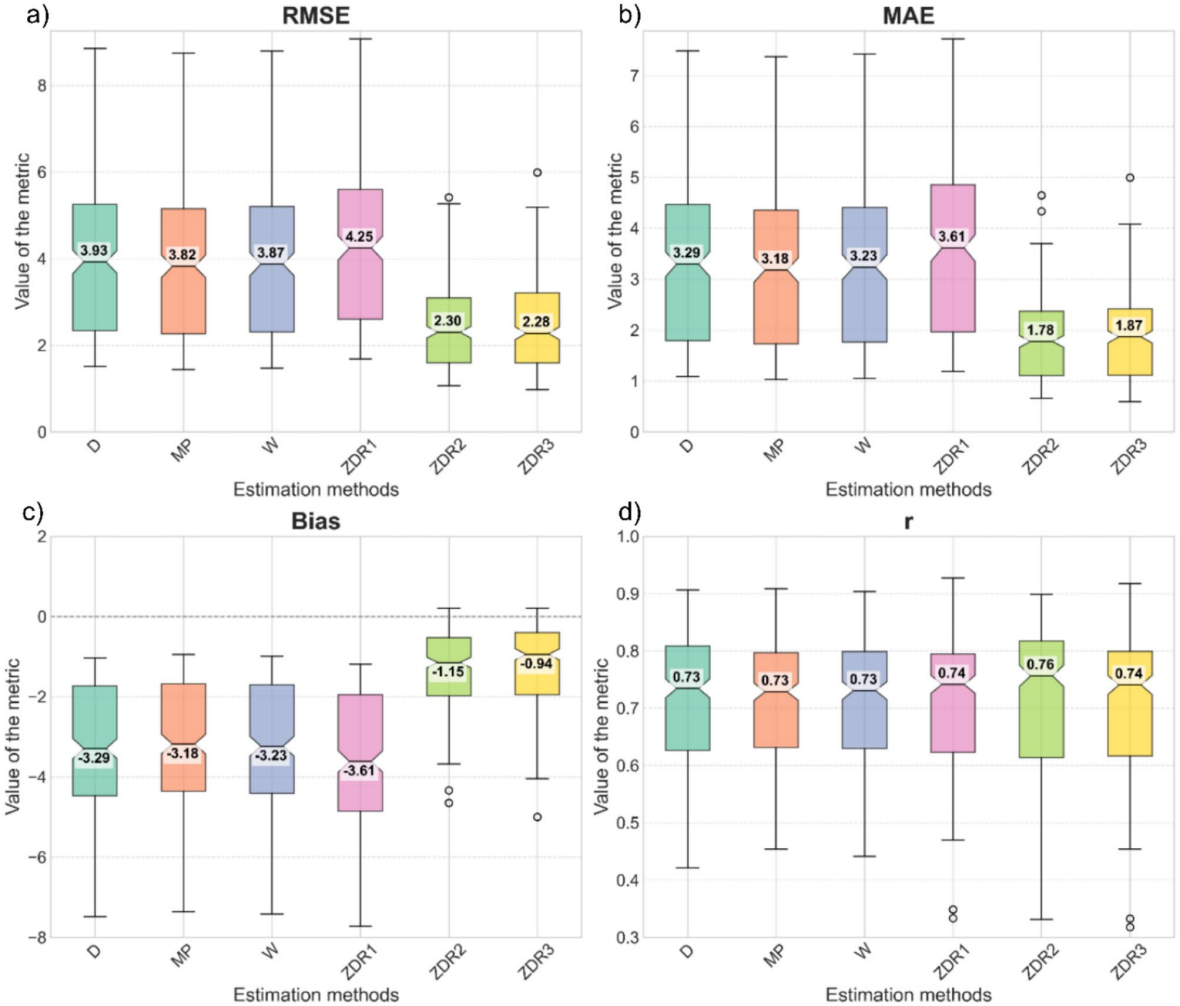
Distribution of statistical metrics for the analyzed rainfall estimation methods. (**a**) RMSE, (**b**) MAE, (**c**) Bias, (**d**) Pearson correlation coefficient (*r*).

Six methods were applied to derive precipitation totals: three classical empirical Z–R relationships (Marshall–Palmer, Wexler, and Doumoulin–Cogombles) and three polarimetric relationships incorporating the ZDR parameter (Table 2). The performance of the methods was evaluated using the statistical metrics RMSE, MAE, Bias, and the Pearson correlation coefficient *r* (Table 3). Radar-derived precipitation estimates were validated against measurements from 21 rain gauge stations (Table 1). The comparative results are presented in Fig. 5, which shows the distribution of errors for each rainfall estimation method.

Fig. 5 presents the distribution of RMSE, MAE, Bias, and r values for the six rainfall estimation relationships (three empirical Z–R and three polarimetric Z–ZDR–R) relative to the rain gauge measurements. The lowest median RMSE was obtained for the ZDR3 relationship (2.28 mm), with a similar value observed for ZDR2 (2.30 mm). The MP, D, W, and ZDR1 relationships exhibited notably higher RMSE values (3.82–4.25 mm) and a substantially larger interquartile range (approximately 2.90 mm), indicating lower stability under diverse terrain conditions. In contrast, the ZDR2 and ZDR3 methods showed a smaller interquartile error range (approximately 1.40 mm), confirming their higher stability and reduced variability compared with the classical Z–R relationships. Despite the generally high agreement, several outliers were observed, associated with rainfall underestimation at the Kamienica station (682 m a.m.s.l.), likely resulting from partial beam attenuation due to orography, which may have contributed to reduced precipitation detection.

All Z–R relationships exhibited higher RMSE values compared with the polarimetric ZDR2 and ZDR3 methods, confirming the beneficial impact of incorporating additional information on drop shape for improving rainfall intensity estimates. The larger errors associated with the Marshall–Palmer, Wexler, and Doumoulin–Cogombles relationships (mean RMSE of approximately 3.87 mm) may result from their calibration for lowland environments, which is not fully suitable for estimating intense precipitation in mountainous terrain.

The ZDR2 and ZDR3 methods exhibited lower and more stable RMSE values compared with the classical Z–R relationships, indicating their potentially higher usefulness in areas with complex orography. To assess the statistical significance of differences among the estimation methods, the Kruskal–Wallis test (two-sided, α=0.05) was applied, followed by Dunn’s post-hoc test with Bonferroni correction. The Kruskal–Wallis test indicated significant overall differences (*p*<0.05); however, the post-hoc pairwise comparisons revealed no statistically significant differences between any method pairs after correcting for multiple comparisons (all *p*≥0.02). The ZDR2 and ZDR3 methods did not differ significantly from each other (*p*=1.0) or from the empirical relationships, despite yielding the lowest numerical error values. A similar pattern was observed for MAE, confirming the consistency of the performance assessment. The MAE values for ZDR2 and ZDR3 were 1.78 mm and 1.87 mm, respectively.

The Bias values indicate a general underestimation of radar-derived precipitation relative to the rain gauge measurements. The smallest Bias values were obtained for the ZDR2 and ZDR3 methods (−1.15 mm and −0.94 mm, respectively). For the remaining relationships, Bias ranged from −3.61 mm to −3.18 mm. The Bias for ZDR3 was approximately 70% lower than that of the operationally used Marshall–Palmer relationship. The improved performance of the polarimetric relationships results from their ability to account for hydrometeor shape, which helps reduce underestimation during intense and long-lasting precipitation events characterized by larger drop sizes.

The correlation coefficient *r* ranged from 0.74 to 0.76 for all examined methods, confirming a strong linear relationship between the radar-derived precipitation estimates and the rain gauge measurements.

Overall, the polarimetric relationships, particularly ZDR2 and ZDR3, provided more precise and consistent rainfall estimates, especially during intense precipitation events characterized by large drop sizes. The highest accuracy was obtained at station S2 using the ZDR3 method with data from the GSA radar (RMSE=0.98 mm, MAE=0.77 mm, and Bias=-0.67 mm), demonstrating the potential for highly accurate precipitation retrievals when the Z–ZDR–R relationships are appropriately parameterized.

## Influence of terrain elevation on estimation error

**Fig. 6:**
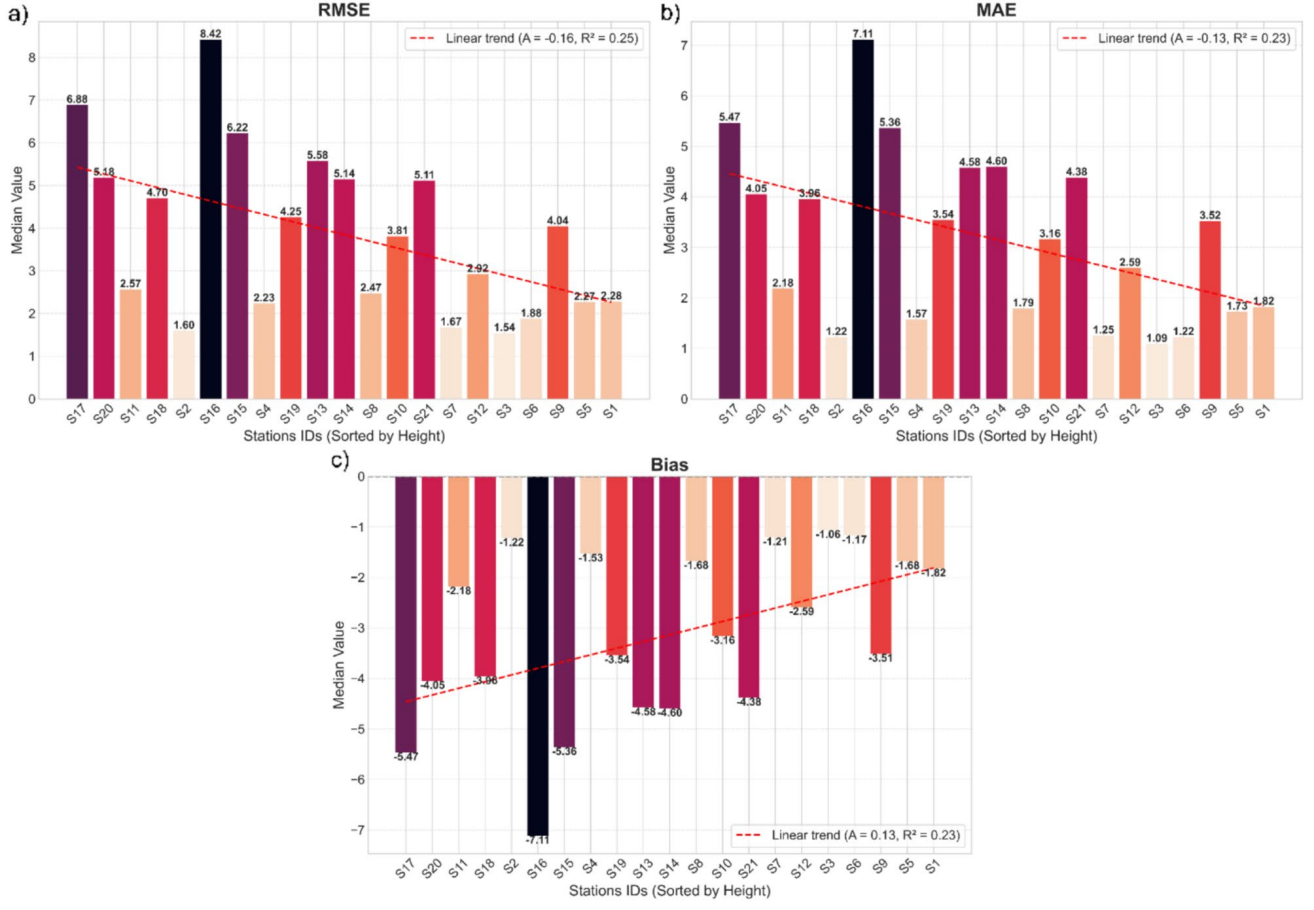
Median values of the statistical metrics for the meteorological stations, ordered by elevation above mean sea level (from highest to lowest), with an accompanying linear trend line. (**a**) RMSE, (**b**) MAE, (**c**) Bias.

The validation of radar-derived precipitation data was performed using measurements from 21 rain gauge stations. Figure 6 presents the distributions of RMSE, MAE, and Bias for all rain gauge stations, arranged according to their elevation above mean sea level.

Figure 6 shows the RMSE, MAE, and Bias values calculated for the hourly radar-derived precipitation totals in comparison with the rain gauge measurements. The trend line indicates that RMSE and MAE decrease with decreasing station elevation, while Bias shows reduced underestimation at lower elevations. This demonstrates that radar estimation accuracy is higher at lower-altitude stations. The lower RMSE and MAE values observed at these locations suggest a reduced influence of orographic effects and weaker signal attenuation, resulting in more representative radar measurements.

The coefficients of determination (*R*^2^) for the fitted linear trends are 0.25 for RMSE and 0.23 for both MAE and Bias, indicating a moderate yet noticeable relationship between station elevation and error magnitude. These *R*^2^ values confirm that orographic factors and radar beam geometry have a substantial influence on precipitation estimation accuracy, although they are not the sole determinants of the observed errors.

Despite the clear general tendency, individual stations show local deviations from the trend. For some stations, the errors are noticeably higher or lower than would be expected based on the overall relationship. The largest errors were recorded at stations S16 and S17, both located above 650 m a.m.s.l. For these stations, RMSE reached 8.42 mm and 6.88 mm, MAE was 7.11 mm and 5.47 mm, and Bias was −7.11 mm and −5.47 mm, respectively, indicating the strongest underestimation among all analyzed locations. The smallest errors, irrespective of the estimation method applied, were obtained at station S3, situated at 364 m a.m.s.l. (RMSE=1.54 mm, MAE=1.09 mm, Bias=-1.06 mm).

The Bias values confirm the general tendency of radar-derived precipitation totals to be underestimated relative to rain gauge measurements, particularly in mountainous areas. The variability in error magnitudes may result from local orographic conditions, radar beam attenuation, and the limited vertical resolution of the radar data. The analysis indicates that terrain elevation and the associated radar beam propagation geometry have a significant impact on precipitation estimation accuracy, both through beam blockage effects and increased signal attenuation in regions with complex orography.

## Spatial variability of errors

**Fig. 7:**
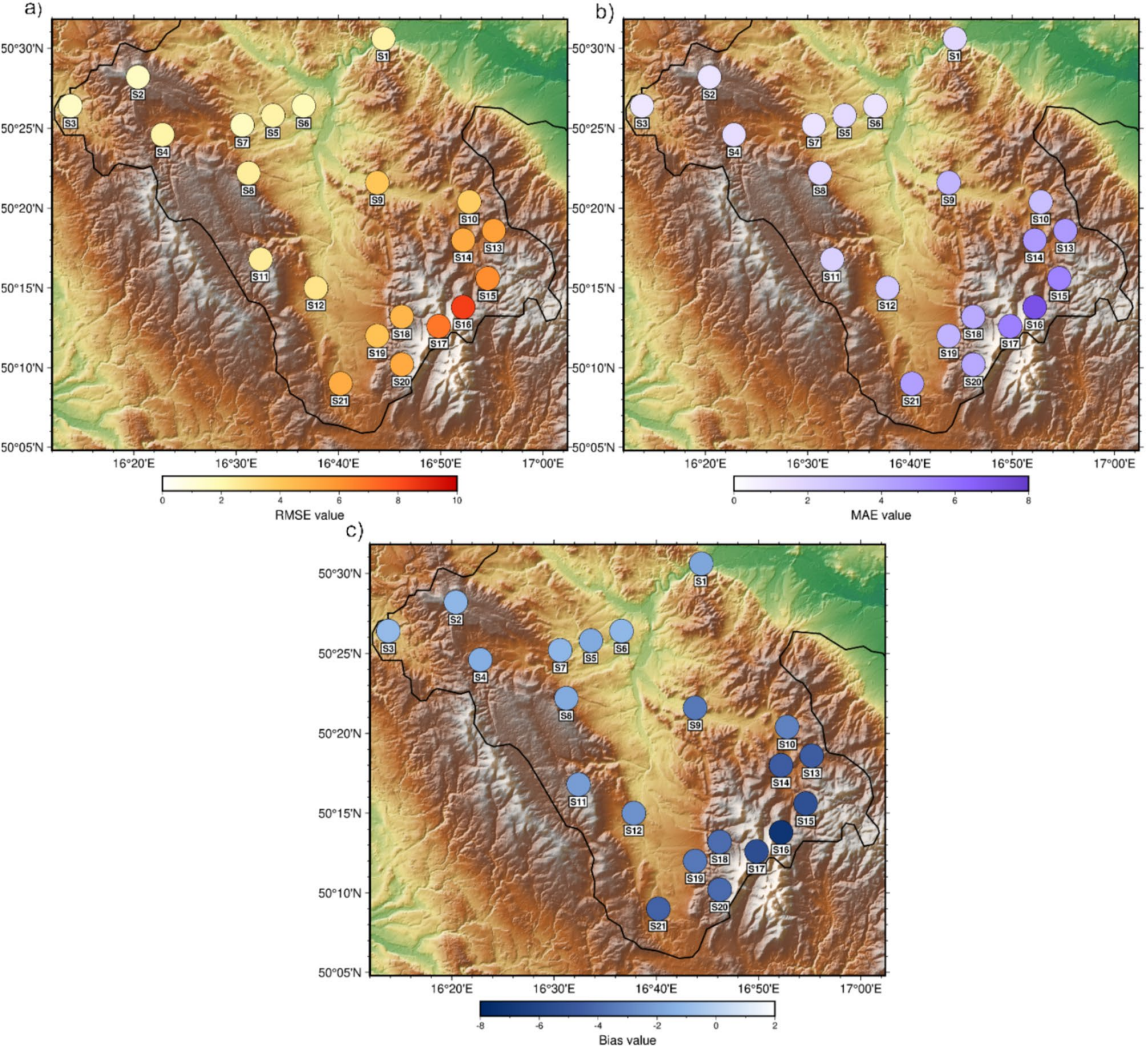
Maps of statistical error values calculated for the rain gauge stations: (**a**) RMSE, (**b**) MAE, (**c**) Bias.

To assess the quality of radar-derived precipitation data relative to point-based rain gauge measurements, a spatial analysis of three statistical parameters: RMSE, MAE, and Bias were conducted. Error values were calculated for each measurement station and presented in Fig. 7 with consideration of the local orography, allowing for the identification of spatial error patterns across the study area. This analysis enables an evaluation of how terrain morphology influences radar data quality and the performance of precipitation estimation methods under varying topographic conditions.

The spatial analysis shows that the largest RMSE and MAE values occur in mountainous areas, particularly in regions with substantial terrain elevation differences. The highest error values were obtained for station S16 (RMSE=8.42 mm, MAE=7.11 mm, Bias=-7.11 mm), located within a mountainous massif where geometric constraints and radar beam attenuation have the strongest impact on measurement quality. Figure 7 reveals a clear clustering of the highest error values around this station and its surroundings, consistent with the mechanism of radar beam shielding by mountain ranges. Under such conditions, the radar cannot sample the lower layers of the atmosphere where precipitation processes frequently occur, leading to underestimation of rainfall totals and increased Bias values.

In areas with less complex terrain, the errors are substantially lower, confirming greater stability of radar detection when appropriate estimation methods and height levels are applied. The smallest errors, regardless of the estimation method, were recorded at station S3 (RMSE=1.54 mm, MAE=1.09 mm, Bias=-1.06 mm), where the radar beam is not obstructed by orography, allowing for accurate precipitation detection.

Orographically forced upward motion enhances precipitation in the lower atmospheric layers, which are often not sampled by the radar; consequently, Bias values differ by as much as 85% between individual stations. The spatial patterns shown in Fig. 7 reveal clear clustering of large errors in the Śnieżnik Massif and within valley areas, whereas the smallest errors occur in regions where detection is supported by overlapping coverage from both radars without beam blockage. This indicates that the spatial distribution of errors is influenced not only by orographic factors and local environmental conditions but also by the parameterization of the applied precipitation estimation methods.

## Comparison of precipitation estimation errors from the two radars as a function of method and data height

**Fig. 8:**
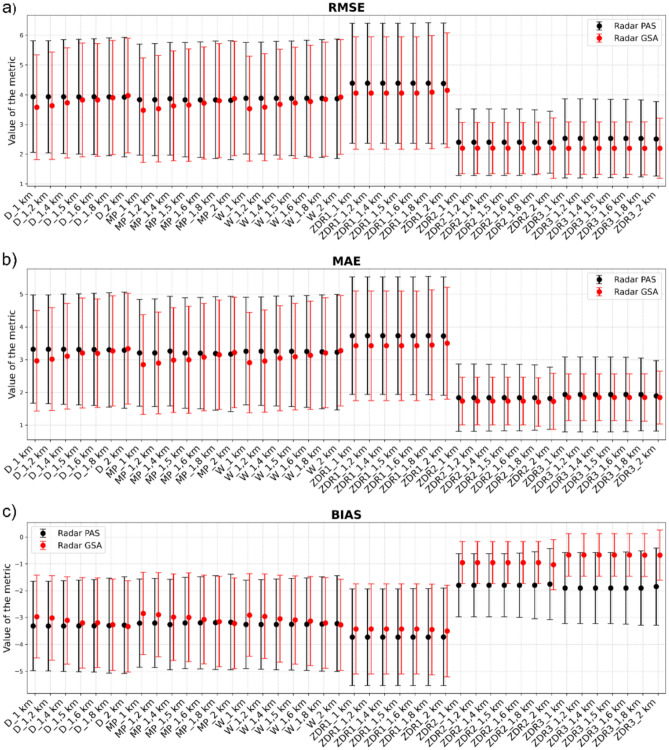
Comparison of mean error values obtained from the PAS and GSA radars for the analysed estimation methods and height levels, including standard deviation (SD): (**a**) RMSE, (**b**) MAE, (**c**) Bias.

To evaluate the performance of precipitation estimation from the two meteorological radars and to determine the influence of data height and distance from the radar, an analysis was carried out using all applied estimation methods. Figure 8 presents the median RMSE, MAE, and Bias values, together with standard deviations, calculated for seven height levels and six methods, separately for both radars (GSA and PAS). This analysis enables a direct comparison of precipitation estimation accuracy between the two radars, which differ in their spatial relation to the study area, and allows assessment of the stability of each method as a function of the input data height.

The analysis confirms that precipitation totals estimated using the polarimetric relationships ZDR2 and ZDR3 show the closest agreement with the rain gauge measurements. Additionally, the results indicate that the GSA radar provides higher accuracy, with RMSE values on average about 0.17 mm lower and MAE about 0.20 mm lower, while Bias is approximately 0.31 mm higher (indicating reduced underestimation) compared with the PAS radar. This demonstrates that, despite the greater distance of the GSA radar from the study area, its precipitation estimates are more accurate, highlighting the effectiveness of polarimetric data in reducing geometry-related measurement errors.

The smallest underestimation was observed for the ZDR2 method applied to the GSA radar data (Bias=-0.002 mm), whereas the largest underestimation occurred for the ZDR1 method using the PAS radar data (Bias=-7.72 mm). The analysis of RMSE standard deviation shows that the most stable results were obtained for the GSA radar combined with the ZDR2 method (SD=0.88 mm), confirming its high repeatability. The greatest variability in error values was observed for the PAS radar using the ZDR1 method (SD=2.08 mm), which may reflect the higher sensitivity of this relationship to temporal changes in drop size distribution.

For the empirical MP, W, and D relationships, the standard deviation ranges from 1.80 to 2.06 mm for RMSE, 1.56 to 1.82 mm for MAE, and 1.57 to 1.84 mm for Bias, indicating moderate stability of the classical Z–R methods. Differences between the height levels do not produce meaningful variations in error magnitude. For the most accurate ZDR3 method, the median RMSE values differ by only 0.01 mm across the analyzed heights, while the standard deviation changes by merely 0.10 mm. This demonstrates that, within the analyzed region, and despite its complex orography, the radar data height has no substantial impact on precipitation estimation accuracy.

The closest agreement with the rain gauge measurements was obtained for the GSA radar using the ZDR3 method, for which RMSE was 2.20±0.90 mm, MAE was 1.84±0.73 mm, and Bias was −0.67±0.81 mm. These results confirm that polarimetric data, particularly when applying the ZDR3 relationship, provide the highest precision and stability in precipitation estimation, regardless of the reference height level.

## Discussion

Although the global Kruskal-Wallis test indicated significant differences between the methods, post-hoc analysis using Dunn’s test with Bonferroni correction did not consistently show statistically significant differences for every pairwise comparison. This can be attributed to the high variance inherent in radar precipitation data over complex terrain, as well as the conservative nature of multiple comparison adjustments. Notably, while the use of ZDR-based relationships (particularly ZDR3) led to a marked numerical improvement in RMSE and Bias, these results should be interpreted as a strong trend rather than a statistically proven universal relationship. However, from a hydrometeorological perspective, the practical significance of these results is substantial. In the analyzed case, the operational significance of the improvement is crucial, as the reduction in precipitation estimation error (69% reduction in Bias and a decrease in RMSE) directly translates into higher quality input data for hydrological models and early warning systems. The stability of RMSE values for polarimetric methods demonstrates their enhanced utility where systematic errors directly impact the reliability of extreme precipitation hazard assessments. Nonetheless, the lack of statistical significance in the rigorous post-hoc tests highlights the need for further research on a larger dataset to confirm the robustness of these relationships in a broader statistical context.

Another technical aspect is the difference in vertical reference frameworks. In this study, classical relationships were compared with polarimetric methods, which operate within different vertical reference frameworks (AGL and AMSL, respectively). Due to the analysis being performed across seven height levels, the geometric locations of the evaluated pixels may exhibit minor discrepancies. Nonetheless, the original settings were retained to assess the practical effectiveness of both approaches under standard operational conditions. This approach reflects the typical configuration in operational radar processing, where each method is applied at its standard reference level. The comparability of the results is maintained by validating all algorithms against the same objective benchmark consisting of a network of 21 rain gauges. Moreover, the vertical offset between AGL and AMSL frameworks is small relative to the radar sampling volume and is thus unlikely to introduce significant systematic bias. Given the vertical extent of the radar sampling volume, these differences do not outweigh the observed performance gains. Consequently, the error reduction observed in the ZDR2 and ZDR3 methods is significant enough that these minor height discrepancies do not impact the overall interpretation of the results or the final conclusions of this work.

Estimating precipitation from radar data using empirical relationships and polarimetric parameters such as ZDR is a well-established approach. Its key advantages include the high temporal frequency and suitability for real-time applications without the need for complex computational models. Unlike artificial intelligence–based methods, empirical relationships do not require extensive training datasets or high computational power. This facilitates their implementation in operational systems, where the use of polarimetric data enhances accuracy, particularly under conditions of variable precipitation microphysics^16,20,37^. In this study, the coefficients for the ZDR2 and ZDR3 equations were derived through local calibration tailored to the analyzed flood event characteristic of the Genoa Low system.

When comparing the results obtained in this study with findings from research conducted in other countries, it becomes evident that continued improvement of the methods and parameters used for radar-based precipitation estimation is essential. Studies carried out in the Shanghai region^37^ confirmed the limitations of classical empirical Z–R relationships and demonstrated that radar-derived rainfall tended to be underestimated (RMSE=3.12 mm, MAE=1.63 mm). These results highlight the need for proper calibration of meteorological radars to ensure that the obtained data are more representative and reliable. Similar conclusions were reported in studies conducted in the state of Missouri^21^, where classical Z–R relationships were compared with formulations incorporating differential reflectivity (ZDR). In that case as well, methods based on polarimetric data exhibited higher accuracy in rainfall estimation, which is consistent with the findings of the present study. Research conducted in the United States^16^ and southern Finland^42^ further emphasized the crucial role of polarimetric data in procedures for estimating precipitation amounts.

Despite their limitations in mountainous regions, radar observations remain an extremely valuable source of information on the spatial distribution and intensity of precipitation. One of their key advantages is high temporal resolution; measurements acquired every 5 minutes enable real-time monitoring of synoptic conditions and early warning of hazardous weather events. The analysis performed for the flood event provided important insights into the operational limitations of radar-based precipitation measurements and allowed for verification of the effectiveness of the proposed approaches. It is important to emphasize that the presented results are based on the analysis of a single, albeit exceptionally intense, precipitation event. This specific episode was intentionally selected to evaluate the algorithms under the most demanding hydrometeorological conditions. While the study focuses on one event, the mathematical framework of the proposed polarimetric relationships and their derivation methodology remain universal. These are based on the physical properties of differential reflectivity ZDR and are applicable across various geographic regions. However, as the empirical coefficients were tailored to the characteristics of the analyzed Genoa Low event, their universal applicability across different synoptic conditions and rainfall types requires further validation and may necessitate recalibration to account for local conditions. While future studies under diverse synoptic conditions will enhance the generalization of these parameters, our results provide robust evidence of the operational advantages of polarimetric methods over classical ones for the analyzed extreme weather scenario. The results obtained in this study can be used to further improve estimation procedures, refine the calibration of meteorological radars, and enhance data quality within hydrometeorological monitoring systems.

## Conclusions

In this study, radar-based precipitation estimation methods were evaluated and validated using rain gauge measurements collected during an extreme flood event. Rainfall totals were derived using the classical empirical Marshall–Palmer, Wexler, and Doumoulin–Cogombles relationships, which describe the link between radar reflectivity (*Z*) and rainfall rate (*R*) at selected heights above ground level. Additionally, three alternative estimation approaches based on polarimetric data, using differential reflectivity (ZDR1, ZDR2, and ZDR3), were proposed and assessed for seven reference levels above mean sea level.

The analysis showed that for the analyzed extreme event, the ZDR3 method (Equation 8) exhibited the smallest errors compared with the other precipitation estimation approaches. The RMSE and MAE values for this relation were 2.28 mm and 1.87 mm, respectively, confirming its high accuracy in determining rainfall intensity under these specific hydrometeorological conditions. The results indicate a general underestimation of radar-derived precipitation relative to rain gauge measurements, which arises from the limitations of observations in mountainous areas - primarily radar signal attenuation, non-uniform precipitation structure, and orographic enhancement of convection. The smallest underestimation was obtained for the ZDR3 method (Bias=-0.94 mm), confirming its high accuracy and suitability for rainfall estimation under complex terrain conditions during intense Genoa Low-type events. The Kruskal–Wallis test (α=0.05) indicated significant global differences between the methods, although post-hoc Dunn’s tests with Bonferroni correction showed that pairwise differences did not reach statistical significance (p≥0.02). This lack of significance in pairwise comparisons does not imply methodological equivalence; rather, it reflects the limited statistical power caused by high natural variability in mountainous precipitation data. From a hydrometeorological perspective, the substantial reduction in systematic bias and the increased stability of RMSE for the ZDR2 and ZDR3 methods provide robust evidence of their operational superiority. These results demonstrate that while physical improvements are practically meaningful for flood forecasting, the high variance inherent in extreme events necessitates a cautious interpretation of conservative post-hoc statistical tests. The Pearson correlation coefficient, which ranged from r=0.73-0.76 for all evaluated methods, highlights the strong relationship between radar-derived and rain gauge observations and confirms the high reliability of the performed estimations.

Radar data were validated using a network of 21 rain gauges, for which statistical error metrics were computed. The results showed that the magnitude of precipitation estimation errors depends on station elevation and local orographic conditions. A systematic increase in error with increasing station altitude was observed. The smallest errors were obtained for the Kudowa-Zdrój station, located at 364 m a.m.s.l. (RMSE=1.54 mm, MAE=1.09 mm, Bias=-1.06 mm), whereas the largest errors were found for the Kamienica station at 618 m a.m.s.l. (RMSE=8.42 mm, MAE=7.11 mm, Bias=-7.11 mm). These results confirm that discrepancies between estimated and observed precipitation increase with elevation and in areas with more complex terrain. The most accurate precipitation representation was obtained using the ZDR3 method for station S2 (RMSE=0.98 mm, MAE=0.77 mm, Bias=-0.67 mm). The smallest underestimation for a single precipitation event was recorded for the ZDR2 method from the GSA radar (Bias=-0.002 mm). These findings indicate the strong potential of radar-based precipitation estimation. Proper parameterization and the selection of an appropriate polarimetric relationship, while accounting for local terrain characteristics, enable high-accuracy rainfall estimation even in mountainous regions with pronounced orographic variability.

A spatial analysis of the obtained error values was also performed. The results showed that stations located in areas with complex orography, particularly on mountain slopes and within valleys, exhibited larger discrepancies between radar-derived and rain gauge precipitation. For stations S1, S5, and S6, situated in areas with gentle terrain, error values ranged from RMSE=1.88–2.28 mm, MAE=1.22–1.82 mm, and Bias=-1.82 to -1.17 mm. In contrast, substantially larger errors were obtained for stations S15, S16, and S17, located within the mountainous massif (RMSE=6.22–8.42 mm, MAE=5.36–7.11 mm, Bias=-7.11 to -5.36 mm). These findings indicate that at greater distances, the radar beam samples higher atmospheric layers. These layers often lie above the main precipitation zone, leading to systematic underestimation. Additionally, orographic lifting enhances precipitation in the lower troposphere, which is not fully captured by the radar. The results confirm that terrain morphology is one of the key factors influencing radar precipitation estimation errors in mountainous regions, particularly during prolonged and intense rainfall events that may lead to flash floods and flooding.

The analysis of data from the two radars showed that measurements from the GSA radar were characterized by smaller errors than those from the PAS radar, despite the latter being located closer to the study area. The precipitation values most consistent with rain gauge observations were obtained from the GSA radar using the ZDR3 relationship (RMSE=2.20±0.90 mm, MAE=1.84±0.73 mm, and Bias=-0.67±0.81 mm). The greatest variability in the estimated precipitation was observed for the PAS radar using the ZDR1 relationship, for which the standard deviation of RMSE was approximately 2.08 mm. These results indicate that in mountainous regions, proximity to the radar is less critical than the beam’s position. Accurate estimation is maintained as long as the beam intersects the atmospheric layer where precipitation occurs. Differences in RMSE values obtained for different height levels were minimal. For the most accurate ZDR3 relationship, these differences were as small as 0.01 mm, with a standard deviation of approximately 0.1 mm, confirming the stability of the results regardless of the reference level. The analysis of RMSE standard deviation showed that the most stable and consistent results were obtained for the GSA radar when using the ZDR2 method (0.88 mm). Overall, the findings confirm that the use of polarimetric indicators and appropriate parameterization of the Z–ZDR–R relationships substantially enhances both the accuracy and stability of precipitation estimation in complex terrain conditions.

The results of this study demonstrated that an appropriate selection of estimation methods and proper parameterization of the underlying relationships can substantially improve the accuracy of radar-based precipitation estimates. The proposed ZDR3 method reduced the RMSE by approximately 37% compared with the operationally used Marshall–Palmer relationship at the 1 km height level. This finding confirms that the incorporation of polarimetric information significantly enhances the quality of precipitation detection and estimation, particularly in areas with complex terrain and during intense rainfall events. However, it should be noted that the empirical coefficients used in the ZDR3 formulation were specifically tailored to the characteristics of the analyzed event.

Reliable radar-based precipitation data constitute a critical source of information for emergency management centers, particularly where high-resolution areal data are essential for effective decision-making. Unlike point-based gauge measurements, radar observations provide the spatial continuity necessary to monitor extreme events across entire catchments. The results of this study demonstrate that integrating dual-polarization data with multi-height analysis aligns with the current operational needs of meteorological services. This approach ensures more effective real-time hazard assessment and provides a validated framework for improving the accuracy of existing operational solutions against modern radar observations.

Further refinement of these methods is possible by extending the analysis to additional cases and different precipitation types to generalize the empirical coefficients. Future research should also focus on employing extended error-correction techniques and incorporating additional polarimetric parameters. Such validation across diverse synoptic situations is essential to establish the universal applicability of the proposed ZDR3 parameters. Such an approach may further enhance the accuracy of operational monitoring and support the development of universal estimation relationships applicable across diverse orographic and climatic environments.

In summary, the conducted analyses confirmed the high effectiveness of polarimetry-based precipitation estimation compared with classical empirical relationships. The ZDR3 relationship emerged as the most effective, exhibiting the lowest errors and the greatest stability under varying conditions. The findings demonstrate that proper parameterization of Z–ZDR–R relationships substantially improves the precision of radar-derived estimates, effectively reducing systematic underestimation. These results constitute a significant contribution to national and international efforts to improve precipitation estimation techniques. Ultimately, the outcomes of this work provide a scientific basis for the calibration of meteorological radars, the enhancement of operational products, and the refinement of emergency management procedures, leading to more accurate flood forecasting and increased hydrometeorological safety.

## Data Availability

The datasets used and/or analyzed during the current study are available from the corresponding author upon reasonable request.
